# Functional roles of lncRNAs and its potential mechanisms in neuropathic pain

**DOI:** 10.1186/s13148-019-0671-8

**Published:** 2019-05-15

**Authors:** Simin Tang, Jun Zhou, Huan Jing, Meijuan Liao, Sen Lin, Zhenxing Huang, Teng Huang, Jiying Zhong, HanbingWang

**Affiliations:** 1 Department of Anesthesiology, The First People’s Hospital of Foshan, Foshan, 528000 Guangdong Province China; 2 Sun Yet-sen University, Guangzhou, 510000 Guangdong Province China; 3 ZunYi Medical University, ZunYi, 563100 China

**Keywords:** lncRNAs, Neuropathic pain, Spinal cord injury, Peripheral nerve injury, Central nerve injury

## Abstract

Neuropathic pain (NP) is ranked as one of the major forms of chronic pain and emerges as a direct consequence of a lesion or disease affecting the somatosensory nervous system. Despite great advances into the mechanisms of NP, clinical practice is still not satisfactory. Fortunately, progress in elucidating unique features and multiple molecular mechanisms of long non-coding RNAs (lncRNAs) in NP has emerged in the past 10 years, suggesting that novel therapeutic strategies for pain treatment may be proposed. In this review, we will concentrate on recent studies associated with lncRNAs in NP. First, we will describe the alterations of lncRNA expression after spinal cord injury (SCI) and peripheral nerve injury (PNI), and then we illustrate the role of some specific lncRNAs in detail, which may offer new insights into our understanding of the etiology and pathophysiology of NP. Finally, we put special emphasis on the altered expression of lncRNAs in the diverse biological process of NP. Recent advances we summarized above in the development of NP may facilitate translation of these findings from bench to bedside in the future.

## Background

Neuropathic pain (NP) has become a worldwide health problem, and its most widely accepted definition is pain caused by a lesion or disease of the somatosensory system [1]. Up to 7–8% of the European population is affected, and in 5% of persons it may be severe [2]. Due to the growing aging global population, increased incidence of diabetes mellitus, and improved survival from cancer after chemotherapy, the incidence of NP has increased [3]. NP can develop after nerve injury, including central nerve injury (CNI) and peripheral nerve injury (PNI), when harmful changes occur in injured neurons and along nociceptive and descending modulatory pathways in the central nervous system (CNS) [4]. Multiple organs and systems are involved in the mechanism of NP, such as the dorsal root ganglia (DRG), spinal cord, and brain [5–7]. In addition, significant changes in neurotransmitters and other molecules, receptors, channels, and signaling pathways are critical to the development of NP at these levels [8, 9]. Although these specific mechanisms of NP have been well described in some previous reviews [3, 10, 11] and considerable knowledge has been gained, it is still complex and difficult to illuminate thoroughly. Fortunately, during the last decades, microarray and high-throughput sequence technology have been widely used to screen genetic alterations at the transcriptome level, which has helped us to identify the differentially expressed genes (DEGs) in the progression of NP [12].

Long non-coding RNAs (lncRNAs) are defined as transcribed RNA molecules, with a length of longer than 200 nt, having no or very low protein-coding potential [13]. According to the genomic location, lncRNAs can be classified into five types: sense, antisense, bidirectional, intronic, and intergenic [13]. Many lncRNAs are implicated in gene-regulatory roles, such as chromosome dosage compensation, imprinting, transcription, translation, splicing, cell cycle control, epigenetic regulation, nuclear and cytoplasmic trafficking, and cell differentiation [14]. According to the characteristics of molecular action, the mechanism of lncRNAs can be further condensed and simplified into four major archetypes: signal, decoy, guide, and scaffold. These four archetypes are interrelated, not mutually exclusive, which are critical to their eventual biological function [15]. To date, it has been reported that lncRNAs are frequently aberrantly expressed and have functional effects in the pathogeny of various human diseases. In a word, lncRNAs may emerge as predictive, prognostic, diagnostic, and therapeutic biomarkers in the future [16]. However, despite recent advances in lncRNAs that have progressed rapidly, the functions of most lncRNAs are still unclear and require more research.

In recent years, the roles and related mechanisms of miRNAs in NP and chronic pain have been well reviewed [17–19]. Although a significant number of researchers examining the crucial role of lncRNAs in NP has been conducted recently, the causal role of lncRNAs in NP has not been expertly summarized. Thus, in the present review, we will summarize the following: (1) expression changes of lncRNAs after CNI and PNI, (2) functional roles of lncRNAs in NP, and (3) altered expression of lncRNAs in biological NP process. This review may lead to breakthroughs in our understanding of NP and provide perspective for diagnostic and therapeutic strategies for NP.

## Expression changes of lncRNAs after CNI and PNI

Changes in the expression of lncRNAs in response to CNI and PNI have been reported. As the leading cause of CNI [3], SCI is attracting considerable interest as the main model exploring expression changes of lncRNAs. Here, we describe the alterations of lncRNA expression after SCI and PNI, which may help to understand the pathogenesis of NP.

### 1.1. SCI

Epidemiological data show that 30–50% of SCI patients have NP, which is one of the most common complications of SCI [20]. More recently, numerous studies have shown that lncRNAs are highly diversified after SCI (Table. 1). For example, Ding et al. [21] found that few changes in lncRNA expression levels were noted 1 day after injury, and significant differential changes in lncRNA expression peaked 1 week after SCI and subsequently declined until 3 weeks after injury. In another study, Zhou et al. [22] used microarray analysis and found that 772 lncRNAs were identified as changed in a rat model for 2 h after SCI. These studies showed that the changes in lncRNA expression have effects on some fundamental processes of SCI physiopathology and may also be equally important in the pathogenesis of NP. However, due to the limited instruments in this field, there are relatively few researches focused on the genetic alterations of SCI. Further studies can focus on exploring the accurate instruments and the specific functional roles of these DEGs.

**Table 1:** The differential expression profile of lncRNAs after nerve injury

SCI model			Methods	LncRNA expression changes	Ref.
Animal	Model	Level			
Male ICR mice (20–25 g, 6–8 weeks)	Contusion SCI model	T 10	Microarray	1dpo: 164 up, 181 down	21
				3dpo: 212 up, 290 down	
				7dpo: 326 up, 565 down	
				21dpo: 141 up, 40 down	
Adult female SD rats (200–230 g)	contusion SCI model	T10	Microarray	2hpo: 528 up, 244 down	22
Adult male ICR mice (male, 8 weeks)	SNL	L5	Microarray	10 dpo: 366 up, 145 down	23
Balb/c mice (male, 8 weeks)	STZ-induced DNP model	L4/L5	Microarray	42dpo:1026 up, 455 down	24
SD rats (180–220 g)	SNI	L4-L6	Transcriptomic analysis	7dpo: 86 known, 26 novel DE lncRNAs	25
Adult male C57BL/6 mice (male, 8 weeks)	SNL	L4	RNA sequencing	6dpo:944 DE (most of them are lincRNAs)	5
Adult male SD rats (250-280 g)	SNI	sciatic nerve	RNA sequencing	1dpo: 35 up, 59 down	26
				3dpo: 44 up, 135 down	
				7dpo: 25 up, 101 down	
				14dpo: 15 up, 129 down	
Adult male SD rats (250-280 g)	SNI	sciatic nerve	RNA sequencing	14dpo: 15 up, 129 down	27
Adult male ICR mice (male, 8 weeks)	SNL	L5	Microarray	10dpo:23 up, 55 down(T-UCRs)	28

*SD rats* Sprague-Dawley rats, *ICR mice* Institute of Cancer Research mice, *dpo* days post-operation

### 1.2. PNI

Changes in gene expression profiles in different animal models were observed after PNI (Table. 1). Jiang et al. [23] used a gene microarray method and found that 366 lncRNAs were upregulated and 145 lncRNAs were downregulated in the spinal dorsal horn of spinal nerve ligation (SNL) model mice. Differentially expressed lncRNAs and 493 differentially expressed mRNAs were then integrated with bioinformatics, and it was speculated that 35 differentially expressed lncRNAs may participate in the formation of NP by affecting the Toll-like receptor signaling, calcium signaling, and peroxisome proliferator-activated receptor signaling pathways. Similarly, Du et al. [24] used microarray analysis and identified 1481 differentially expressed lncRNAs, including 1026 upregulated and 455 downregulated lncRNAs, in the L4, L5 spinal dorsal horns of DNP mice. Mao et al. [25] used a transcriptome-level deep sequencing and found 86 known and 26 novel differentially expressed lncRNA genes in L4-L6 DRGs after spared sciatic nerve injury. Wu et al. [5] used RNA-sequencing technology and found that the expression of 944 ncRNAs had significantly changed in the L4 DRG in the SNL model, most of which were lncRNAs. Furthermore, Zhou et al. [26, 27]. used RNA second-generation sequencing analysis to analyze the gene expression profiles in spared nerve injury (SNI) rat model. It was revealed that 35, 44, 25, and 15 lncRNAs were upregulated at 1, 3, 7, and 14 days, and 59, 135, 101, and 129 lncRNAs were downregulated. In addition, transcribed ultraconserved regions (T-UCRs), as highly conserved lncRNAs, are involved in the regulation of transcription and posttranscriptional gene expression. Jiang et al. [28] found that the expression of T-UCRs in the L5 spinal dorsal horn of SNL mice changed significantly compared with sham-operated mice. Among the 78 altered T-UCRs, 23 T-UCRs were upregulated by more than 1.5-fold, and 55 T-UCRs were downregulated to less than 0.5-fold after SNL.

## Functional roles of lncRNAs in NP

The differential expression of lncRNAs is increasingly recognized as a hallmark feature in various diseases, especially in cancer [29], and this is also applicable to NP. Although there are few studies on the functional roles of lncRNAs in NP mechanisms at present, some studies have examined some characterized lncRNAs and described their functional roles in NP-associated processes, such as transcription interference and epigenetic regulation. Here, we will highlight the emerging functional roles of lncRNAs in NP (Table.2).

**Table 2:** LncRNAs axis associated with neuropathic pain

LncRNAs	Target gene	TFs or relevant factors moleculars	Model	Ref.
Kcra2 AS RNA	Kcna2 mRNA	–	SNL and CCI rat model	31,32,33,34
XIST	miR-137	TNFAIP1	CCI rat model	36
XIST	miR-150	ZEB1	CCI rat model	37
XIST	miR-34a	–	CCI rat model	38
XIST	miR-544	STAT3	CCI rat model	39
XIST	miR-154-5p	TLR5	CCI rat model	40
XIST	miR-494	STAT3	CCI rat model	41
uc.48+	P2X7 receptor	p-ERK1/2	Diabetic rat model	47
uc.48+	P2X7 receptor	p-ERK1/2	TN rat model	52
uc.48+	P2X3 receptor	–	Diabetic rat model	48
NONRATT021972	P2X7 receptor	–	Diabetic rat model	46,50
NONRATT021972	P2X3 receptor	ERK1/2, p-ERK	Diabetic rat model	49
BC168687	P2X7 receptor	NO	DNP rat model	53
BC168687	P2X7, TRPV1receptor	TNF-α, IL-1β/p-ERK, p-p38	DNP rat model	54
MRAK009713	P2X3 receptor	–	CCI rat model	55
DGCR5	miR-330-3p	PDCD4	CCI rat model	56
MALAT1	miR-206	ZEB2	CCI rat model	57
LINC00657	miR-136	ZEB1	CCI rat model	58
NEAT1	miR-381	HMGB1	CCI rat model	59
FKBP51	–	–	CCI rat model	60
CCAT1	miR-155	SGK3	bCCI rat model	61

*SNL* spinal nerve ligation, *bCCI* bilateral chronic constriction injury, *CCI* chronic constriction injury, *DNP* diabetic neuropathic pain, *TN* trigeminal neuralgia, *TFs* transcription factors, *NGF* nerve growth factor, *BDNF* brain-derived neurotrophic factor

### 2.1. Kcna2 antisense RNA: cis-acting repressor

*Kcna2 antisense RNA* (*Kcna2 AS RNA*) is a 2.52 kb cis-encoded lncRNA expressed in mammalian DRG neurons, and most of its sequence is complementary to Kcna2 RNA, a voltage-gated K+ channel. This natural antisense transcript suppresses the expression of the Kcna2 gene, decreases the expression levels of the K+ channel, and thus alleviates the NP. Kcna2 is highly expressed in rats, whereas *Kcna2 AS RNA* is only expressed in 20% of DRG neurons under physiological conditions [30, 31]. Recent research indicated that nerve injury induced an increase in myeloid zinc-finger gene1 (MZF1) [31], histone-lysine N-methyltransferase 2 (known as G9a) [32], and DNA methyltransferase (DNMT3a) [33, 34], which can enhance the transcription of *Kcna2 AS RNA*, and a decrease in Kcna2 mRNA and protein expression in the DRG. The increased expression of *Kcna2 AS RNA* specifically and selectively inhibited the expression of Kcna2 mRNA via extensive overlap of their complementary regions, including the transcription and translation inhibition sites, leading to reduced expression levels of the membrane Kcna2 channel and an increased number of action potential and neuronal excitability in DRG neurons, which produced spinal cord central sensitization and NP symptoms [30, 31]. Blocking *Kcna2 AS RNA* effectively alleviated the hyperalgesia behavior of NP rats. Generally, *Kcna2 AS RNA* may act as a cis-acting repressor to be an endogenous trigger in NP development and maintenance.

### 2.2. XIST: “miRNA sponge” or “ceRNA”

**Fig. 1:**
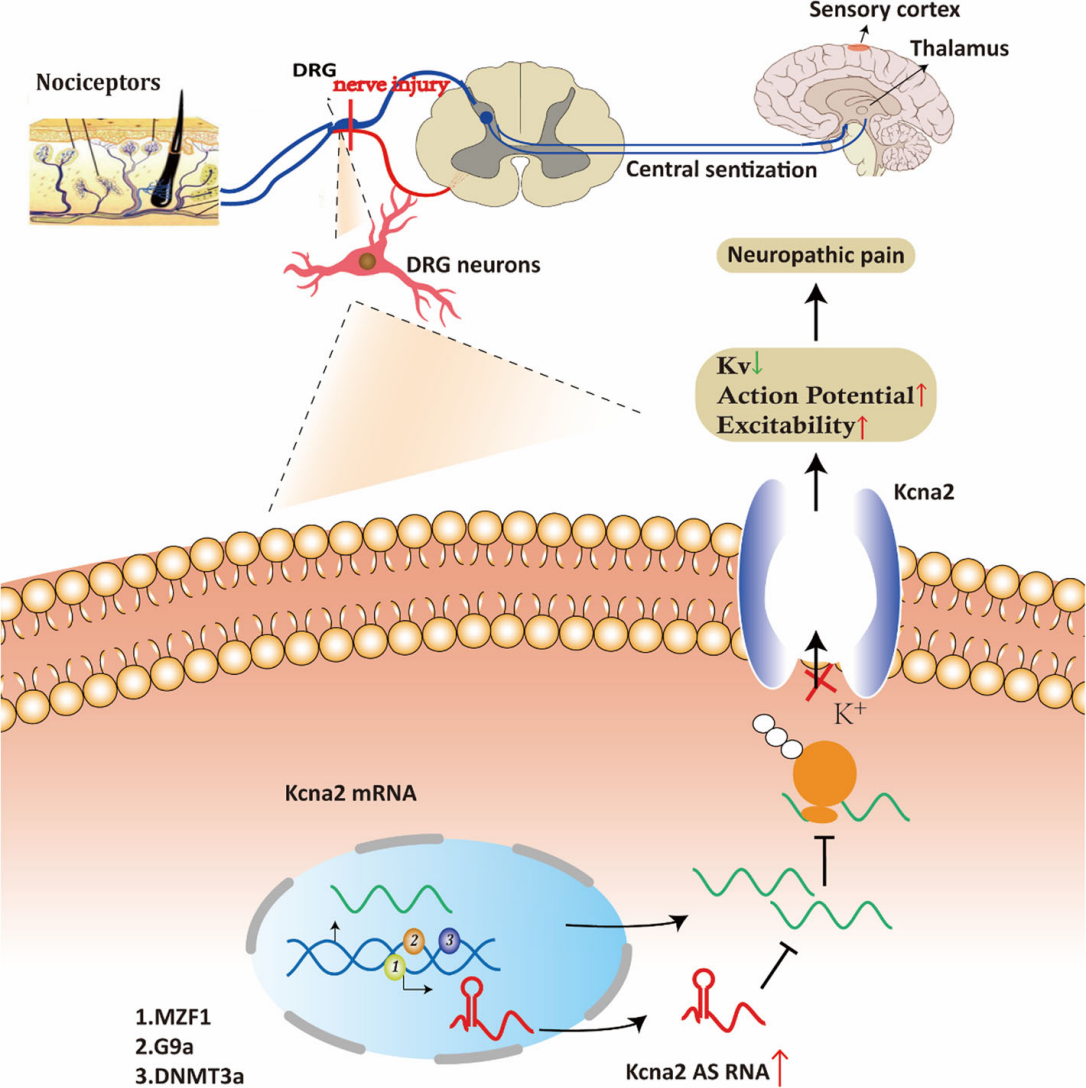
Role of Kcna2 AS RNA under NP condition. Under NP condition, the increased expression of Kcna2 AS RNA can specifically and selectively inhibits the expression of Kcna2 mRNA, which then leads to the decreased expression level of Kcna2 channel and increased action potential and neuronal excitability in DRG neurons, producing central sensitization and ultimately alleviating the symptoms of NP.

XIST is a 17 kb spliced, polyadenylated transcript that acts as a major effector of X-inactivation center process in female mammals. The expression of XIST has to be tightly controlled, involving the X-inactivation center, a cis-acting region and many other lncRNA genes that evolved to XIST from protein-coding ancestors through pseudogenezation and loss of coding potential [35]. Previous studies showed that the expression of miRNAs in NP can be deregulated by a range of mechanisms, including copy number alterations and epigenetic silencing. It is speculated that XIST may act as a natural “miRNA sponge” to reduce the expression levels of miRNA (Fig. 1b). In recent studies, XIST appeared to function as miRNA sponge sequestering miR-137, miR-150, miR-544, and miR-154-5p, then regulating the expression of relevant binding proteins or the release of inflammatory cytokines that accelerated NP progression [36–40]. In addition, Gu et al. [41] demonstrated that XIST effectively becomes a miRNA sponge for miR-494, also acting as a competitive endogenous RNA (ceRNA), and contributed to neuronal apoptosis through the downregulation of AKT phosphorylation in the SCI model. Of note, Botros et al. showed that miR-34a can regulate XIST under inflammation directly and through pro-inflammatory transcription factor YY1 in complex regional pain syndrome (CRPS) patients^44^. These findings implied that XIST may regulate neuroinflammation to maintain or develop NP through sponging miRNAs.

### 2.3. LncRNAs mediate P2X receptors

**Fig. 2:**
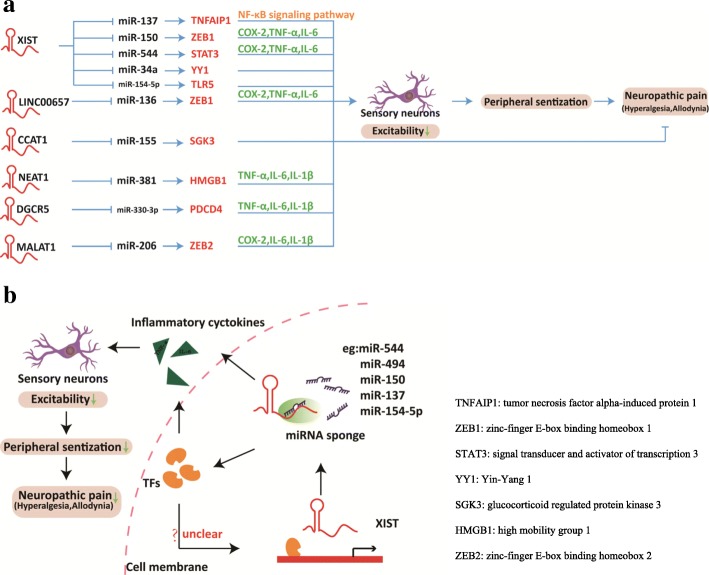
Schematic of lncRNA-miRNA interactions in CCI rat models. **a** Ingenuity pathway analysis of differentially expressed lncRNAs mediated miRNA in CCT rat models. **b** XIST functions as “miRNA sponge” to reduce the expression levels of miRNA in NP, it prevents TFs (such as TNFAIP1, ZEB1, STAT3) from microRNA-mediated suppression, or directly decrease the release of inflammatory cytokines, thus alleviating the symptoms of NP. How the TFs affect the transcription of XIST is unclear. TNFAIP1 tumor necrosis factor alpha-induced protein 1, ZEB1 zinc-finger E-box binding homeobox 1, STAT3 signal transducer and activator of transcription 3, YY1 Yin-Yang 1, SGK3 glucocorticoid-regulated protein kinase 3, HMGB1 high mobility group 1, ZEB2 zinc-finger E-box binding homeobox 2

LncRNAs can regulate P2X receptor expression, mostly evidenced in various disease states. ATP is an endogenous ligand of purinergic P2X receptors (P2XRs), which is abundant in neuronal and glial cells. It is released upon cell stress, damage, or stimulation, thereby activating P2XRs that are present in the sensory afferent endings to produce pain [42, 43]. Specifically, P2X7 receptors function as ligand-gated ion channels [44], while P2X3 receptors are preferentially expressed in DRG neurons and are upregulated under neuropathic, inflammatory, and visceral pain hypersensitivity conditions [45]. Recent studies have shown that the expression of lncRNAs was abnormally altered in the serum of diabetic patients and diabetic rats when pathologic pain occurs, indicating that lncRNA-mediated P2X receptors may be a suitable target for analgesic drugs (Fig. 2). For instance, small inhibitory RNA to lncRNA NONRATT021972 and uc.48+ can downregulate rat P2X7 and P2X3 receptor expression in NP, which reduced the release of inflammatory cytokines, inhibited the excitability of DRG neurons, and reduced mechanical and thermal hyperalgesia in T2DM rats [46–50]. In a clinical study, researchers found that NONRATT021972 has abnormally increased expression in the DRG and serum of diabetic patients and diabetic rats, and the data indicated that NONRATT021972 was positively associated with neuropathic pain scores of type 2 diabetes [51]. However, there are few studies on the clinical relevance and translation potential of these findings, and we suggest future studies can concentrate on clinical practice. Additionally, a recent study showed that uc.48+ participate in pain transmission in trigeminal neuralgia, the most common NP in the facial area, via upregulating expression of P2X7 receptor and furthermore enhance the phosphorylation of ERK1/2 [52]. Similarly, BC168687 siRNA inhibited the expression of P2X7 receptors and influenced the pathological process of DNP [53, 54]. In another study, MRAK009713 directly interacted with the P2X3 protein expressed in the CCI rat model and potentiated P2X3 receptor function [55]. Collectively, these studies indicate that some lncRNAs are upregulated in disease states to increase P2X receptor expression, which indicates that lncRNAs mediate P2XR purinergic sensory pathways to the spinal cord dorsal horn and may be another major mechanism of NP.

### 2.4 Other lncRNAs

Aside from the above lncRNAs, many other lncRNAs regulate gene expression at the transcriptional and posttranscriptional levels by regulating epigenetics and interactions with chromatin-modifying complexes which then affect the genetic mechanisms of NP (Fig. 1a). For instance, Peng et al. showed that lncRNA DGCR5 alleviates NP through sponging miR-330-3p and regulating PDCD4 in CCI rat models [56]. Chen et al. revealed that inhibition of lncRNA MALAT1 ameliorates CCI-induced NP in rats via modulating miR-206 and ZEB2 [57]. Shen et al. [58] indicated that LINC00657 can regulate ZEB1 expression by acting as a sponge of miR-136 in NP development. Xia et al. [59] indicated that NEAT1 induced NP development in CCI rats via regulating the miR-381/HMGB1 axis. Yu et al. [60] found that silencing of FKBP51 alleviated the mechanical pain threshold and inhibited DRG inflammatory factors and pain mediators through the NF-κB signaling pathway. Dou et al. [61] revealed that overexpression of CCAT1 partially alleviated the pain threshold by acting as a sponge for miR-155 through targeting SGK3. SGK3 is an important inflammatory signaling protein that plays a key role in signaling pathways and cellular phosphorylation cascade [62]. However, we still have many challenges, and it will take work to explore the function and biological relevance of lncRNAs.

## 3. Altered expression of lncRNAs in biological NP process

Recent studies have highlighted genetic alterations involved in the progression of NP, and aberrant lncRNA expression participates in NP by disrupting major biological processes, such as glial cell activation, affecting signaling pathways, or altering the expression levels of ion channels. Thus, additional studies are needed to explore the specific mechanism of lncRNA in NP. Hence, we will briefly describe the expression of lncRNAs in the biological process of NP to deepen our understanding of NP, which may steer future research and guide clinical practice.

### 3.1. LncRNAs involved in activation of glial cells

#### 3.1.1. Microglia

Glial cells comprise over 70% of the total cell population in the CNS and are subdivided into astrocytes, oligodendrocytes, and microglia [63]. Microglial cells are known as resident macrophages in the CNS, which derive from primitive macrophages in the yolk sac. Microglia can proliferate, become hypertrophic and activated after peripheral inflammation and nerve image, and then secrete molecules such as IL-1, IL-6, and TNF-α that sensitize sensory neurons in the dorsal horn, contributing to the development of NP [63–65]. Although several recent studies have associated lncRNA expression with microglia, the direct effects of lncRNAs on establishing microglia have not been determined and await elucidation. For instance, lncRNA MALAT1 promotes a high glucose-induced inflammatory response of microglial cells via provoking MyD88/IRAK1/TRAF6 signaling in a cerebral injury model in diabetic rats [66]. Intriguingly, it was pointed out that MyD88 is involved in the development of immune system and chronic pain [67]. Thus, further study can focus on how lncRNAs interact with immune pathways under pain condition. MALAT1 also contributes to the inflammatory response of microglia following SCI by modulating the miR-199b/IKKβ/NF-κB signaling pathway [68]. In conclusion, MALAT1 is closely related to the inflammatory reaction of microglia cells in a variety of pathological and physiological circumstances, such as brain injury and SCI described above. Similarly, lncRNA fantom3_F730004F19 may be involved in microglia-inducing inflammation via the TLR signaling pathway [69]. LincRNA-Cox2 plays vital roles in the activation of NLRP3 during inflammation and autophagy in macrophages and microglia [70]. The contribution of spinal cord microglia activation to central sensitization and pain processes has emerged as a new concept [64]. Thus, an understanding of the role of lncRNA in microglia cells may enable its use as a prognostic factor or even a therapeutic target.

#### 3.1.2. Astrocytes

A few lines of evidence have shown that lncRNA expression may contribute to the progression of NP in astrocytes, the major glial cell type within the CNS, which is thought to maintain exaggerated pain in NP [71]. Astrocytes can proliferate and produce pro-inflammatory cytokines and chemokines after PNI [63]. For instance, recent studies found that astrocytes and microglia in the ipsilateral spinal cord dorsal horn were activated after SNL-induced NP, and the expression profiles of lncRNAs and mRNAs were significantly changed, assessed using microarrays [23, 26]. Similarly, Zhang et al. [72] showed an upregulation of the expression levels of inflammatory cytokines secreted by microglia and astrocytes in the spinal cord dorsal horn at 10 days after SNL. Han et al. [73] found that overexpression of H19 induced the activation of astrocytes and microglia and the release of pro-inflammatory cytokines in the hippocampus. Although studies have not directly associated lncRNAs with astrocytes in NP, the available research suggested that lncRNAs may be involved in the progression of astrocyte activation, affecting NP by regulating pro-inflammatory cytokines or signaling pathways. Knowledge of the regulatory lncRNAs and their roles in astrocytes of NP is still limited.

#### 3.1.3. Oligodendrocytes

Oligodendrocytes produce myelin for axonal insulation in the CNS [63]. Mechanical and cold hypersensitivity was induced by genetic oligodendrocyte ablation in naive mice, and perturbation of oligodendrocyte functions that maintain axonal integrity can lead to central neuropathic pain [74, 75]. Recent work points to the role of lncRNAs in oligodendrocyte precursor cell (OPC) differentiation from neural stem cells, myelination, and remyelination in the CNS, and scientists have established the Sox10-Venus mouse system to analyze the differentiation and multipotency of murine OPCs, which will be helpful for in-depth research [76]. He et al. [77] used transcriptome reconstruction to reveal a dynamic network of lncRNAs in oligodendrocytes, which indicated that stage-specific myelination control by a lncOL1/Suz12 complex in the CNS. Li et al. [78] showed that lnc158 positively regulated the transcription level of NFIB mRNA and contributed to an enhanced induction of oligodendrocytes. However, similar to astrocytes, the direct evidence of lncRNA regulation of oligodendrocytes in the mechanism of NP is still poor.

### 3.2. LncRNAs involved in signaling pathways of NP

**Fig. 3:**
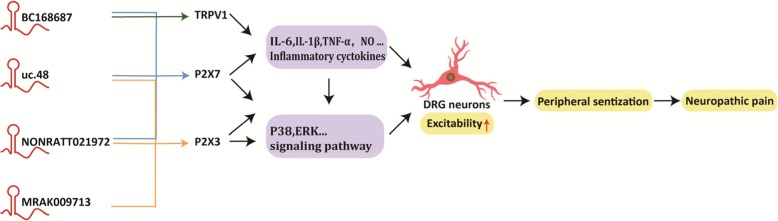
LncRNAs modulate pain signal transmission by mediating P2X receptors. As can be seen from the Figure, overexpression of lncRNAs can upregulate the expression of P2X7 and P2X3 receptor in DRG neurons, induce the release of inflammatory cytokines, or activate the pain-related signaling pathways, thereby activating the excitability of DRG neurons and ultimately promoting NP.

Numerous signaling pathways play core roles in the mechanism of pain at different levels, including in DRG neurons, spinal cord neurons, and the brain. As a result of recent studies, lncRNAs, the mediators in signaling pathways, have been acknowledged to play vital roles in the transduction or inhibition of signaling actions (Fig. 3). For instance, studies have shown that the MAPK and NF-κB signaling pathways regulated by lncRNAs may be responsible for the majority of inflammatory mediator-signaling actions within nociceptive neurons [54, 60, 79, 80]. The ERK pathway, as a branch of the MAPK signaling pathway, is another main pathway that indicates a relationship between lncRNAs and NP [61, 80]. In addition, Zhang et al. [81] used Kyoto encyclopedia of genes and genomes (KEGG) pathway enrichment analysis and pathway network analysis to disclose differentially expressed genes and activated signaling pathways in association with SCI-induced NP and found that 209 pathways changed significantly; among them, the most significantly activated one is the MAPK signaling pathway. Similarly, numerous studies have also shown that lncRNAs are highly differentially expressed in the spinal cord of mice after SCI, and researchers integrated these differentially expressed lncRNAs and mRNAs using bioinformatics, speculating that these lncRNAs may participate in the formation of NP by affecting various signaling pathways, such as the JAK-STAT signaling pathway, p53 signaling pathway, and Toll-like receptor signaling pathway [21–23, 26–28]. Overall, lncRNAs may function like a “molecular switch” to toggle between signaling pathways, thus regulating the underlying mechanisms of NP. We suggest that the characteristics of lncRNAs should be examined within signaling pathways, exploring the specific mechanism of NP in future work.

### 3.3. LncRNAs alter NP-related ion channels

In general, NP is initiated by opening sensory voltage-dependent ion channels within nociceptive terminals in response to damaging stimuli of sufficient strength, such as inflammatory factors [82]. Voltage-gated ion channels associated with pain perception are placed on neurons that are essential for the transmission and modulation of noxious or potentially tissue-damaging stimuli [83]. It has been reported that lncRNAs alter ion channel expression involved in NP, although most of these findings are not in depth, it still provides novel insight into the molecular mechanisms of NP. For instance, *Kcna2 AS RNA*, a new native lncRNA, is an antisense transcript that suppressed the expression of Kcna2 mRNA and protein, led to reduced expression levels of the membrane K+ channel that then increased the number of action potentials and neuronal excitability in DRG neurons resulting in spinal cord central sensitization and symptoms of NP [30, 31]. Likewise, purinergic P2X7 receptors, a nonselective cation channel permeable to Ca2+, K+, and Na+ is activated by ATP or pro-inflammatory cytokines. This receptor is engaged both in inflammation and in NP. Researchers have shown that small inhibitory RNA to NONRATT021972, uc.48+, and BC168687 can inhibit the expression of P2X7 receptor expression in a DNP rat model and modulate ion channel expression, thereby alleviating the symptoms of NP [46, 47, 50, 53, 54]. In addition, researchers used bioinformatics and found that calcium ion transport was the second most significant biological process of differentiate expressed lncRNAs [24]. Certainly, specific mechanisms of lncRNAs and their association with ion channels are still elusive, and further research is needed.

### 3.4. Potential mechanisms of autophagy and apoptosis regulated by lncRNAs in NP

Autophagy and apoptosis are biological cellular processes that impact cellular homeostasis and direct cell fate and have recently been implicated in several human diseases, including NP and inflammation [70, 84, 85]. More recently, studies have shown that lncRNAs have a close relationship with various diseases [16], and we can predict that the mechanisms of autophagy and apoptosis regulated by lncRNAs may play important roles in NP. On the one hand, autophagy participates in microglial cell functions, which are associated with the pathophysiology of NP [86]. Autophagy can also regulate inflammatory signaling pathways, such as the NF-κB pathway, by modulating the release of pro-inflammatory cytokines, which is also related to the development of NP [87, 88]. On the other hand, Wang et al. [89] indicated that MALAT1 exerted neuroprotective effect in a rat model of spinal cord ischemia-reperfusion injury by regulating miR-204, which plays a pivotal role on the processes of SCI physiopathology and may also be equally important in the pathogenesis of NP. All these studies suggested that autophagy and apoptosis may be regulated by lncRNAs in NP. Although the research is still in its infancy, we can still predict that the association of lncRNAs with autophagy and apoptosis in NP may be a new experimental research method for understanding NP in the future, but much work needs to get done.

## Conclusions

The lines of evidence described above indicate that functional roles of lncRNAs maintain and develop NP by binding to mRNA, sponging to miRNAs, or binding to pain-related genes. In addition, aberrant lncRNA expression disrupts major biological processes, then promoting the progression of NP. Therefore, studying the specific role and mechanisms of lncRNAs in NP will provide valuable insight into the molecular basis of NP, which may lead to new therapeutics and diagnostics. However, throughout the discussion above, we found that there are still some challenges faced in current studies. Compared to protein-coding mRNAs, lncRNAs exist on average at a lower abundance, frequently reside in the nucleus, are more tissue-specific and lack strong conservation, suggesting that they are non-functional in diverse diseases. Most of the current NP research aims at the development of highly conserved lncRNA molecules. Thus, how to conduct effective bioinformatics predictions on the spatial structure and function of such low conservative lncRNA molecules and how to study their role in human pathological pain and its functions are all challenges.

Moreover, in addition to the already known mechanisms of lncRNAs in NP, further mechanisms of lncRNAs in the molecular or genetic aspects of NP still need to be explored. Here, we suggest that future research could focus on the outlined questions, as listed below: (1) Although the specific molecular mechanisms of lncRNA have been elaborated in multiple physiological and pathological processes quite exhaustively, direct in-depth proof of the mechanisms related to NP needs further development. For instance, the role of lncRNA molecules in NP is mainly concentrated in the peripheral nerve DRG and SDH, while the pain-related brain regions on the spinal cord are not involved. (2) The annotated lncRNAs that we have discussed may be just the tip of the iceberg, and the large number of unannotated lncRNAs may contain more important functions in the pathogenesis of NP. (3) There is difficulty in studying the NP mechanism itself, due to the need to obtain clinical specimens; however, exosomes can provide a new perspective for our clinical studies. The role of lncRNA in the exosomes of cerebrospinal fluid is worthy of attention. (4) Can we explore the diagnostic or therapeutic potential of lncRNAs to promote human health? Despite the fact that this has been an elusive goal, promising translational efforts are not far behind.

## Abbreviations

AP: Action potentials
BDNF: Brain-derived neurotrophic factor
CCI: Chronic constriction injury
CNI: Central nerve injury
CNS: Central nervous system
DNMT: DNA methyl transferase
DNP: Diabetic neuropathic pain
DRG: Dorsal root ganglia
GO: Gene ontology
HMGB1: High mobility group 1
ICR mice: Institute of Cancer Research mice
KEGG: Kyoto Encyclopedia of Genes and Genomes
lncRNAs: Long non-coding RNAs
NGF: Nerve growth factor
NP: Neuropathic pain
OPC: Oligodendrocyte precursor cell
P2XR: Purinergic P2X receptor
PNI: Periphery nerve injury
PNS: Peripheral nervous system
SCI: Spinal cord injury
SD rats: Sprague-Dawley rats
SGC: Satellite glial cells
SGK3: Glucocorticoid-regulated protein kinase 3
SNARE: Soluble NSF attachment protein receptor
SNI: Spared nerve injury
SNL: Spinal nerve ligation
STAT3: Signal transducer and activator of transcription 3
TNFAIP1: Tumor necrosis factor alpha-induced protein 1
T-UCRs: Transcribed ultraconserved regions
ZEB1: Zinc-finger E-box binding homeobox1
